# Supplementary material for: Single-photon detection using large-scale high-temperature MgB2 sensors at 20 K
Source: Nat Commun. 2024 May 10;15:3973. doi: 10.1038/s41467-024-47353-x (PMC11087534; doi:10.1038/s41467-024-47353-x)
Supplement: Supplementary file 1 — Supplementary Information [file 41467_2024_47353_MOESM1_ESM.pdf]

Supplementary Information for Single-photon detection using large-scale high-temperature MgB_2 sensors at 20 K

Ilya Charaev*,[1,2] Emma K. Batson,[1] Sergey Cherednichenko*,[3] Kate Reidy,[1] Vladimir Drakinskiy,[3] Yang Yu,[4] Samuel Lara-Avila,[3] Joachim D. Thomsen,[1] Marco Colangelo,[1,5] Francesca Incalza,[1] Konstantin Ilin,[6] Andreas Schilling,[2] and Karl K. Berggren*[1]

[1]*Massachusetts Institute of Technology, Cambridge, MA 02139, USA*

[2]*University of Zurich, Zurich 8057, Switzerland*

[3]*Department of Microtechnology and Nanoscience, Chalmers University of Technology, Göteborg SE-41296, Sweden*

[4]*Raith America, Inc., 300 Jordan Road, Troy, NY 12180, USA*

[5]*Department of Electrical and Computer Engineering, Northeastern University, 360 Huntington Ave., Boston,02115, USA*

[6]*Institute of Micro- and Nanoelectronic Systems, Karlsruhe Institute of Technology (KIT), 76187 Karlsruhe, Germany*

Supplementary Note 1 - Superconducting MgB_2 film deposition and characterization

Here we report on the deposition and characterization of superconducting MgB_2 films. Magnesium melts at 650 degrees Celsius and interacts with boron. The latter is produced via the thermal decomposition of gaseous diborane (B_2H_6) supplied in a mixture with hydrogen (5% : 95%) at a flow of 2 sccm with a background hydrogen flow at 400 sccm. The total pressure is 20 Torr. More details on the growth of magnesium diboride thin film can be found in previous studies.[1]

In total, we have deposited more than a dozen films in order to obtain MgB_2 SNSPD devices with the highest critical temperature. To reach this goal, we completed a variety of tasks such as (1) optimization of deposition temperature, (2) gaseous diborane B_2H_6 flow variation, (3) sweeping of process power, and (4) reduction of the deposition rate. In Fig. 1 we summarize the superconducting and transport properties of deposited films. The sheet resistance was measured using a 4-probe technique. The films were characterized in terms of their RT-dependencies in closed-cycle cryostat from 300 down to 3.7 K. The critical temperature for this dataset was recorded at the temperature where $R_{50\mathrm{K}}/2$. The thickness of deposited films was controlled by XRR reflectivity (details below).

Name	R_{300}, Ω/sq.	T_c, K	d, nm	Time, s
**F189A**	13.7	39.3		120
**F190A**	16.3	39.1	11.33	100
**F191**	16.2	37.6	12.49	80
**F192**	36.5	35.5	9.6	60
**F193**	15.3		16.8	60*
**F158A**	15.1	38.85	15.7	140
**F158B**	15	37.95	15.45	140
**F159A**	15.0	37.2		130
**F159B**	19.20	38.1		130
**F159C**	12.73	37.65		130
**F159D**	15.20	38.7	12.1	130
**F180**	14.2	38.8	11.9	

Supplementary Fig. 1. **Superconducting and transport properties of magnesium diboride films** where R_{300} is a sheet resistance at 300 K; T_c - the critical temperature; d - the thickness; Time - the deposition time.

Supplementary Note 2 - Second critical magnetic field

The temperature dependence of the second critical magnetic field B_{c2} was measured by applying an external magnetic field perpendicularly to the film surface. Calculation of the electron diffusion coefficient was accomplished by taking the linear part of the temperature dependence (Fig. 2) of B_{c2}. We used the following expression to estimate the diffusion constant:

$$D = (\frac{-4k_B}{\pi e})(\frac{dB_C2}{dT})^-1 \tag{1}$$

This resulted in the value of the electron diffusion coefficient $D = 0.98$ cm^2/sec.

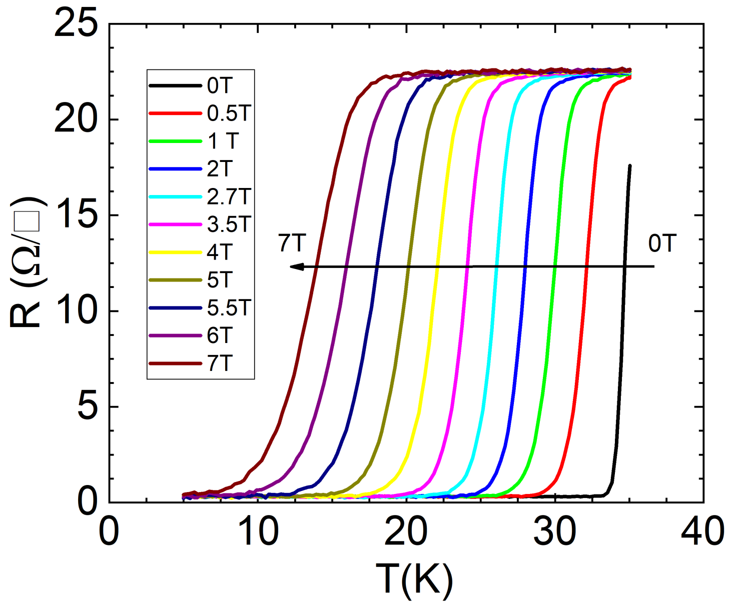

Supplementary Fig. 2. **Second critical magnetic field in magnesium diboride films**

Supplementary Note 3 - Detector geometry

We designed the detectors with various widths and active areas. Fig. 3 shows the summary of detector geometries. While the filling factor was kept the same to prevent current crowding in turns of meander, the active area and the number of squares varied in the range up to 400×400 μm^2 and up to 10200 squares respectively.

Supplementary Note 4 - Switching current

We conducted an analysis of the switching current in devices, which involved performing I-V sweeps for wire widths ranging from 1 to 5 μm at varying temperatures. The results

W, µm	*A*, µm^2	*FF*	N_{sq}
**1**	200×200	0.28	10200
**2**	400×400	0.28	10200
**3**	400×400	0.28	4700
**4**	400×400	0.28	2500
**5**	300×300	0.28	1260

Supplementary Fig. 3. **Summary of microscale wide detector geometry.** W is a width of wires; A - the active area of detector; FF - filling factor; $N_{\rm sq}$ - the number of squares.

of the switching current were plotted in Fig. 4. In general, the switching current does not exhibit a linear relationship with wire width.

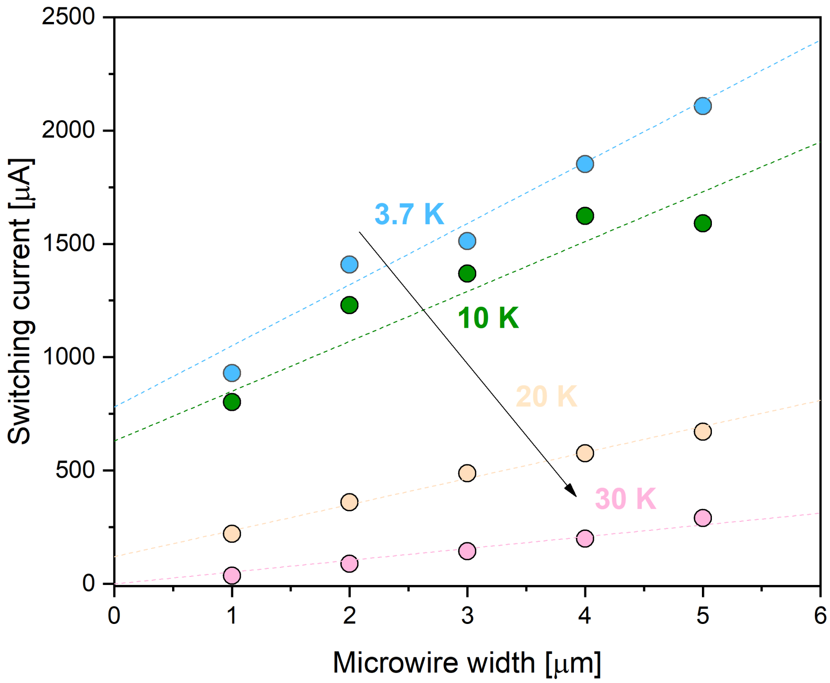

Supplementary Fig. 4. **Switching current** The dependence of the switching current on the device width and a bath temperature.

Supplementary Note 5 - He^+ dose tests for MgB_2 detectors

Before the fabrication of SNSPD out of MgB_2, we analyzed the He^+ beam exposure effects on the superconducting properties of the MgB_2-based films. To this end, we measured the critical current, I_c, of MgB_2 bridges as a function of the He^+ dose (Fig. 5). We found that while 10^{15} ions/cm^2 had practically no effect on I_c, 10^{17} ions/cm^2 resulted in the full suppression of the superconductivity in this material. The dose 5×10^{15} ions/cm^2 caused only mild suppression of I_c but decreases of I_r (Fig. 7) and thus to the enhancement of single-photon sensitivity of MgB_2 detectors. Additionally, we implemented a Monte-Carlo simulation of the interaction of helium ions with MgB_2m(Fig. 6). In our study, damage per ion was estimated by using the SRIM [J. Ziegler, "SRIM and TRIM.] "quick calculations" mode to simulate vacancies in a target material stack resulting from irradiation with 30 keV helium ions. Although SRIM in this mode has some limitations, typically underestimating actual damage per ion,[2] this mode should be sufficient for qualitative comparisons of irradiation damage across different film stacks.

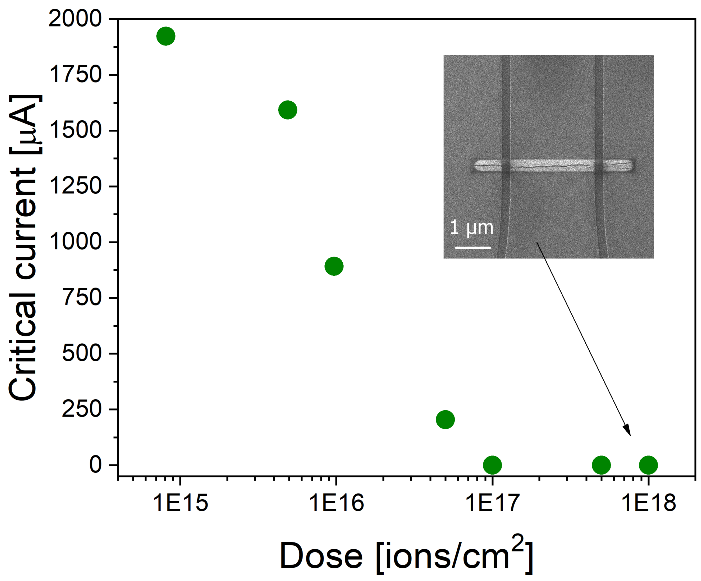

Supplementary Fig. 5. **He^+ dose test for MgB_2 microbridges.** Inset shows the SEM image of the exposed microbridge at the dose of 10^{18} ions/cm^{-2}.

Supplementary Note 6 - X-ray reflectivity measurements

To verify the thickness in our MgB_2 films, we performed the XRR measurements using Rigaku Smartlab with an incident-beam Ge(022) monochromator, which is used for high-resolution XRR on relatively thick films up to 300 nm. The sample was precisely aligned and the measurement conditions were optimized automatically based on sample information. Fig. 8 shows a few examples of taken data for MgB_2 films.

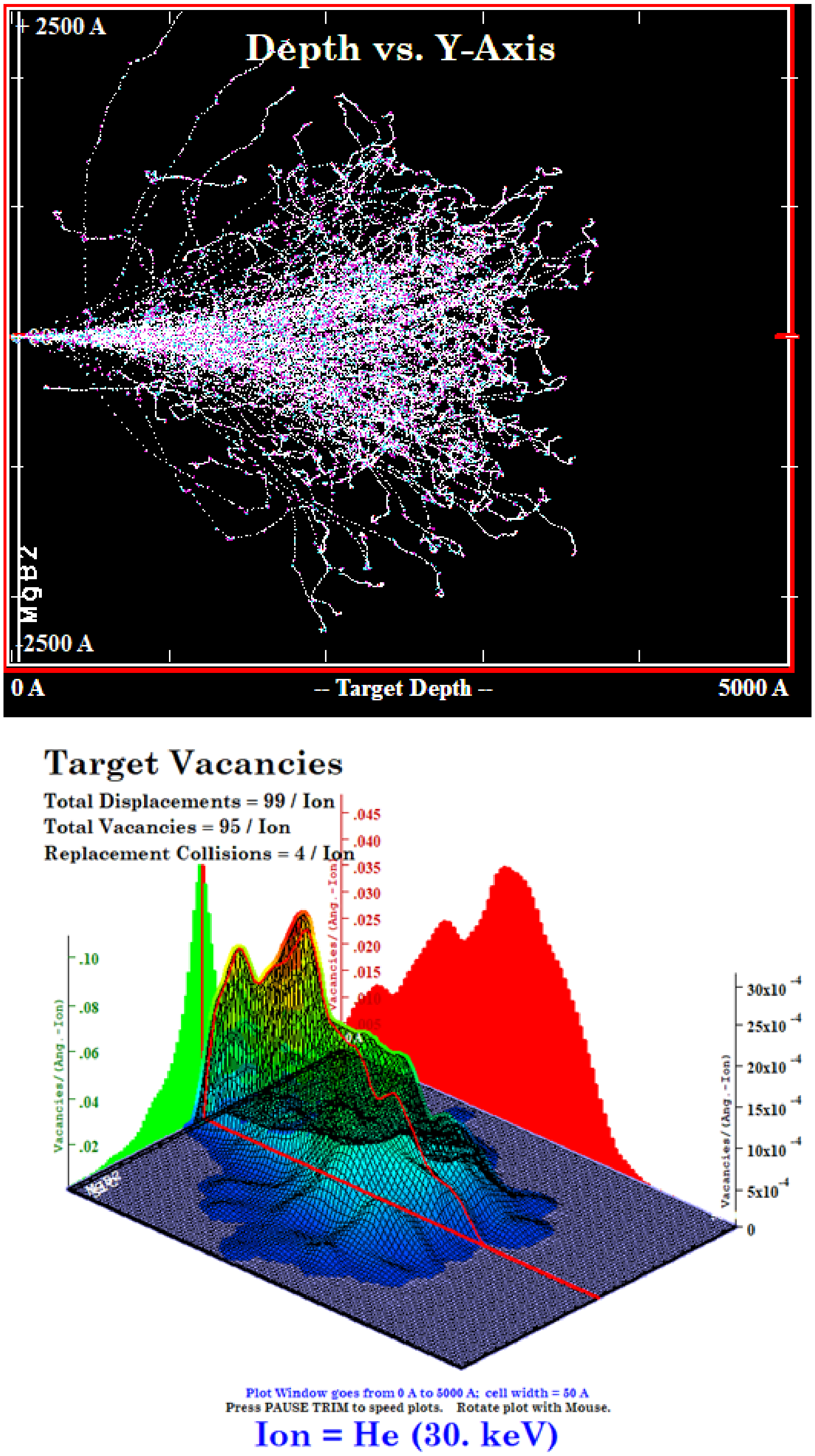

Supplementary Fig. 6. **Simulation of the interaction of 30 keV helium ions with the MgB_2 device.** The results of the Monte-Carlo simulation performed using Stopping and range of ions in matter package (SRIM).

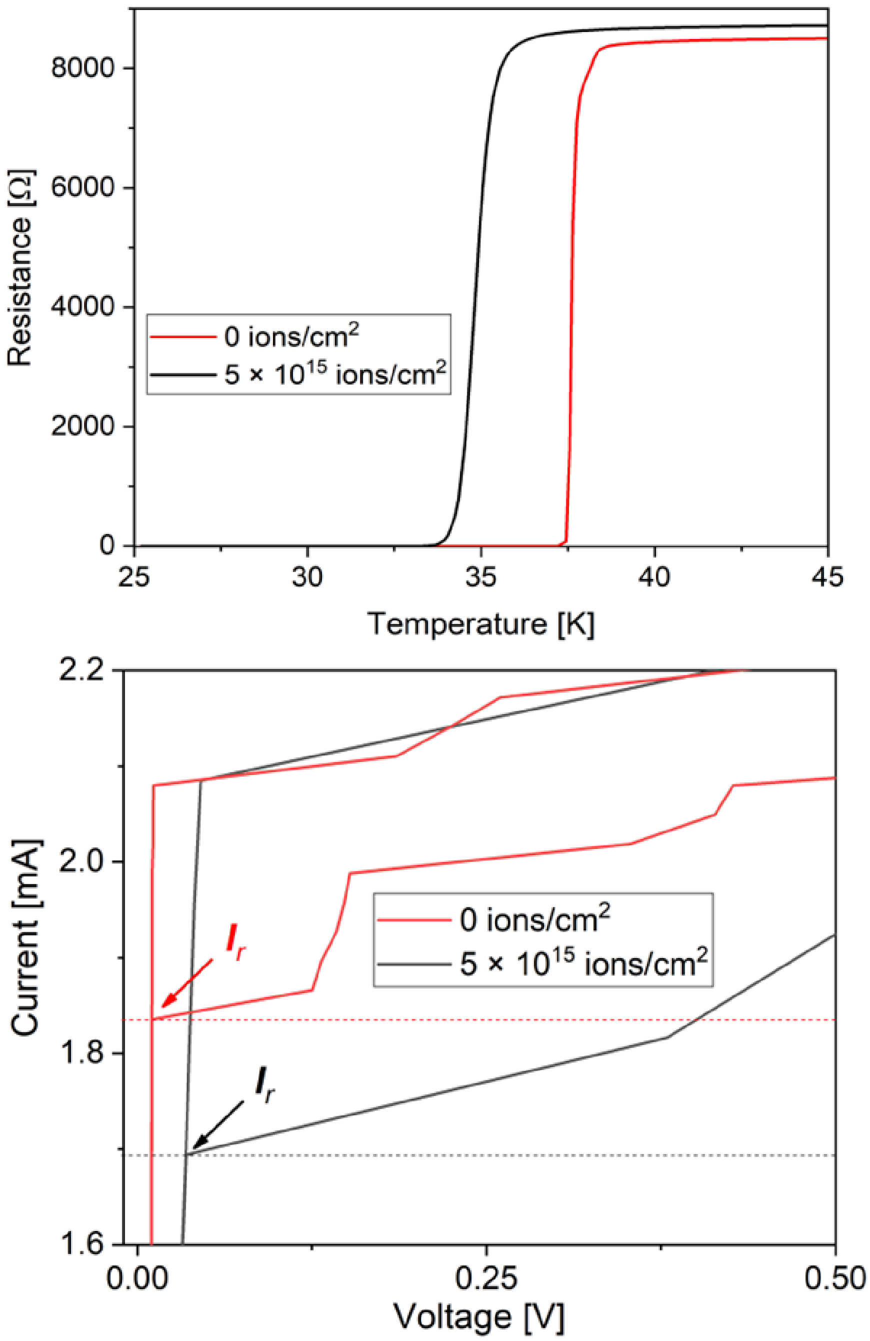

Supplementary Fig. 7. **Effect He^+ irradiation on critical temperature, resistance, switching, and retrapping current.** The resistance as a function of the temperature was measured in DC-bias mode. Current-voltage characteristics taken from 5-μm wide MgB2 device before and after He^+ irradiation at 3.7 K.

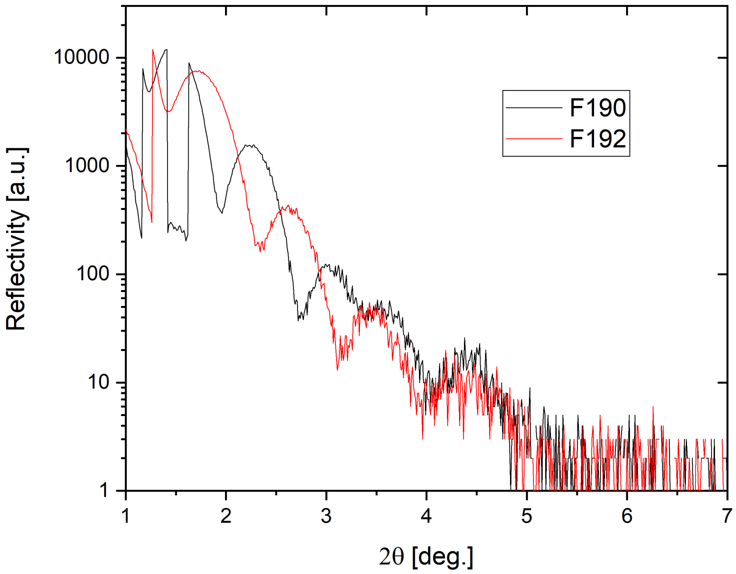

Supplementary Fig. 8. **X-ray reflectivity measurements.** Experimental X-ray reflectivity (XRR) as a function of twice the incident angle for films shown in the legend.

Supplementary Note 7 - Energy Dispersive X-Ray Spectroscopy (EDS)

The material analysis was performed on a film prepared by the same growth process described in Methods. The film showed a magnesium oxide on either side of the MgB_2 film (which is 2-6 nm thick for each layer).

Supplementary Note 8 - Timing jitter

To measure timing jitter, we used an experimental setup with a 1550 nm femtosecond-pulsed laser that was adopted as the single-photon source. The real-time oscilloscope builds a statistical distribution of the arrival times of photon counting pulses. We used the 1 μm wide detector with an active area of 200 by 200 μm^2 (see table in Fig. S3).The distribution typically has an almost Gaussian profile (Fig. 10). We defined the timing jitter as the full width at half maximum (FWHM) of the distribution. We found that the jitter of micro-scale MgB_2 detectors is similar to conventional SNSPD detectors to be 50.3 ps.

Supplementary Note 9 - Estimating detection efficiency

Here we calculate the detection efficiency, DE, of 1 μm wide MgB_2 detector at different temperatures. This can be estimated by accounting for determined $\sim$ 11.5% absorption, α, of thin MgB_2 films on a SiC substrate at 1550 nm wavelength by determination of the impedance function.[3]

$$DE = PCR(\alpha f A_d/A_{beam})^{-1} \tag{2}$$

where A_{beam} is the area of the defocused laser beam incident on the chip, A_d is the total detector area, PCR is the photon count rate, and f is the photon flux. Using experimentally

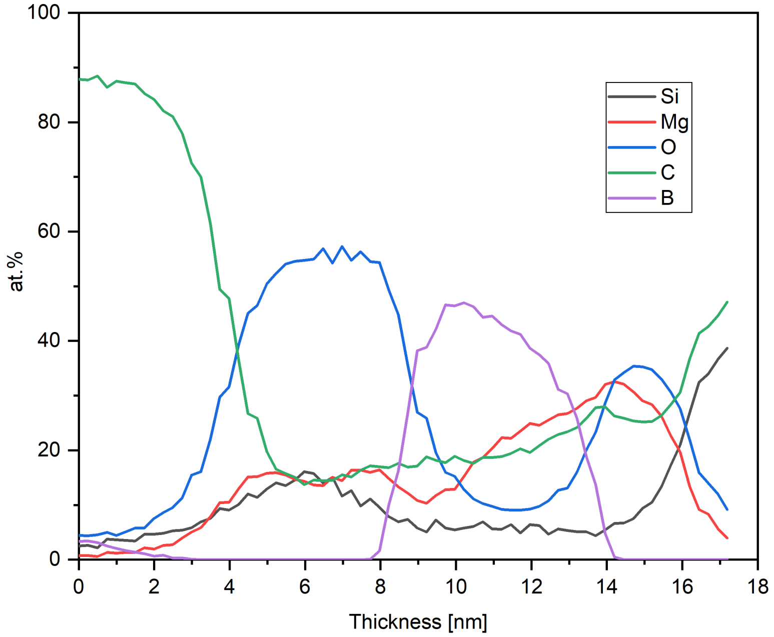

Supplementary Fig. 9. **EDS measurements performed on MgB_2 film prepared by the same growth process as used for the patterned films.** The left-hand side of the graph ($0 - 2$ nm) corresponds to the top of the film with a carbon protection layer from FIB processing, while the right-hand side ($16 - 18$ nm) shows the SiC substrate. The EDS atomic % obtained are not quantitatively accurate for light elements such as B and O, however, are intended to give a qualitative overview of the elemental distribution across the sample thickness.

determined $PCR = 10^3$ s^{-1} for 1 μm wide detector (Fig. 3b) measured for the 40 dB attenuated laser radiation at $\lambda = 1.5$ μm and assuming that the laser beam spot radius is 7.5 mm, visually determined using red laser light fed into the same optical fiber, one obtains $DE \sim 7.6\%$ at 20 K. At 10 and 3.7 K, we find DE exceeding 10%, to be 11.3%, and 14.1% respectively. The discrepancy in values between DE and absorption arises marginally, primarily attributable to the reflection of photons from the holder back to the detector.

However, these estimates are approximate as they do not account for the polarization dependence of meander geometry that can lead to both enhancement and reduction of the apparent efficiency. Moreover, we did not carry out accurate measurements of the A_{beam} and used the lower bound for the laser spot size. In addition, we did not account for variations in the power of the optical light source over a long time. Nevertheless, we note that DE can be further boosted by integrating the MgB_2 detectors into the photonic cavity to enhance light-matter interaction.

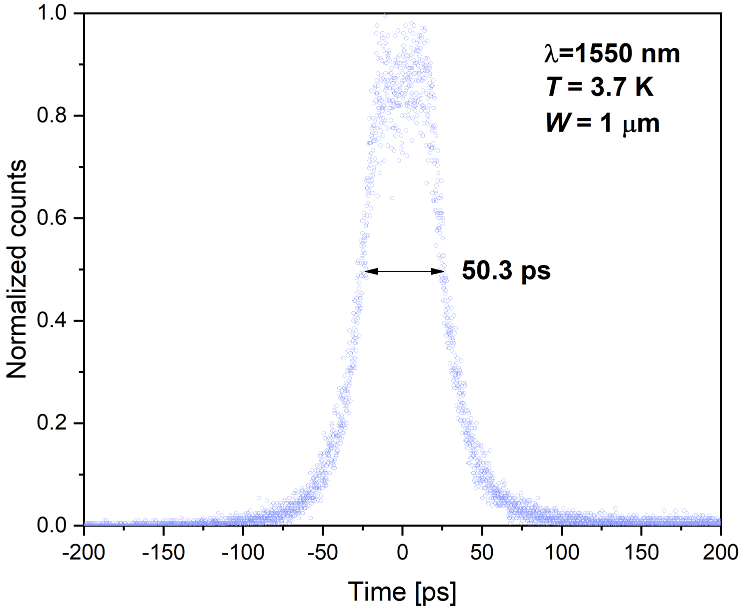

Supplementary Fig. 10. **Timing jitter measurements.** The combination of the detector's normalized instrument response function and the femtosecond laser synchronization signal is represented in a histogram. The jitter of 50.3 ps is indicated by arrows with a sign.

[1] Novoselov, E., Zhang, N. & Cherednichenko, S. Study of MgB_2 ultrathin films in submicron size bridges. *IEEE Transactions On Applied Superconductivity* **27**, 1-5 (2016).

[2] Weber, W. & Zhang, Y. Predicting damage production in monoatomic and multi-elemental targets using stopping and range of ions in matter code: Challenges and recommendations. *Current Opinion In Solid State And Materials Science* **23**, 100757 (2019).

[3] Semenov, A., Günther, B., Böttger, U., Hübers, H., Bartolf, H., Engel, A., Schilling, A., Ilin, K., Siegel, M., Schneider, R., Gerthsen, D. & Gippius, N. Optical and transport properties of ultrathin NbN films and nanostructures. *Phys. Rev. B* **80**, 054510 (2009).
